# Editorial: EBV strategies to counteract the immune response

**DOI:** 10.3389/fimmu.2026.1895384

**Published:** 2026-06-25

**Authors:** Stefano Lazzi, Cristiana Bellan, Paola Chabay, Rita Khoueiry, Lucia Mundo, Paul Murray

**Affiliations:** 1 Department of Medical Biotechnology, University of Siena, Siena, Italy; 2 Pathology Division, Multidisciplinary Institute for Investigation in Pediatric Pathologies (IMIPP-CONICET-GCBA), Ricardo Gutiérrez Children’s Hospital, Buenos Aires, Argentina; 3 International Agency for Research on Cancer (IARC), Lyon, France; 4 Department of Clinical and Molecular Medicine, Sapienza University, Rome, Italy; 5 School of Medicine, Bernal Institute, Limerick Digital Cancer Research Centre and Health Research Institute, University of Limerick, Limerick, Ireland; 6 Department of Pathology, School of Medicine, Royal College of Surgeons Ireland, Busaiteen, Bahrain

**Keywords:** EBV, exhaustion, immune response, latency, lymphoma, traces

The Epstein-Barr virus (EBV), first discovered in 1964 in Burkitt lymphoma, infects over 95% of the global population and is associated with approximately 200,000 cancer cases each year. However, in most people EBV infection does not lead to cancer. Rather, the virus has evolved to coexist with its host by establishing a lifelong, largely asymptomatic infection. The ability of EBV to persist in memory B cells for the life of an individual arises from a balanced interaction with the host immune system, facilitated by mechanisms that promote immune evasion. This Research Topic explores the complex interplay between EBV and the human host, and encompasses molecular mechanisms of viral persistence and immune regulation, as well as the diverse clinical spectrum of diseases ranging from infectious mononucleosis to aggressive malignancies.

In this Research Topic, Sausen et al. demonstrate that EBV promotes the development of an immunoregulatory microenvironment that enables tumour cells to evade host immune surveillance. The authors highlight several mechanisms underlying this process, including manipulation of antigen presentation through the glycine–alanine repeat (GAr) domain of EBNA1, together with the induction of immunosuppressive interleukins and cytokines, and upregulation of inhibitory immune checkpoints such as PD-L1, all of which contribute to impaired T-cell function.

Silva et al. describe EBV as a “mastermind” of immune evasion, employing a highly sophisticated network of latent proteins, including LMP1 and EBNA1, together with virally encoded microRNAs, to manipulate host immune responses. The authors highlight how these viral factors cooperate to inhibit apoptosis, promote cellular proliferation, and disrupt immune surveillance mechanisms mediated by natural killer (NK) cells and cytotoxic T lymphocytes.

To further explore the mechanisms of immune evasion triggered by EBV, Vietzen et al. studied immune responses in EBV-positive lymphoma. They found that a polymorphic LMP1 peptide correlated with the HLA-E*0103/0103 variant, which inhibits NKG2A+ NK cells and promotes the dissemination of EBV-infected tumors. Furthermore, deletions in the KLRC2 gene reduced the protective responses of NKG2C+ NK cells, thereby facilitating tumor spread. Notably, targeting the NKG2A receptor with Monalizumab could restore NK-cell-mediated control, presenting a potential immunotherapeutic option for these cancers.

Desimio et al. demonstrated that Epstein–Barr virus (EBV) modulates susceptibility to natural killer (NK) cell surveillance in a phase-dependent manner by challenging primary NK cells with EBV-positive lymphoblastoid cell lines during both latent and lytic infection. Transition into the lytic phase, driven by expression of the viral immediate-early protein BZLF1, resulted in marked downregulation of HLA-E together with increased expression of ligands for the activating receptor NKG2D, particularly MICB. These changes rendered lytically infected cells significantly more susceptible to NK cell-mediated cytotoxicity. Importantly, BZLF1-positive cells were preferentially eliminated through an NKG2D-dependent mechanism, while blockade of HLA class I molecules further enhanced killing. Interestingly, inhibition of the normally suppressive NKG2A receptor paradoxically reduced NK-cell-mediated lysis, highlighting a specialised role for NKG2A-positive NK-cell subsets, including CD56^bright^ and NKG2A^+^KIR^+^CD56^dim^ populations, in recognising and responding to lytically infected EBV-positive cells. These findings suggest that EBV lytic reactivation transiently exposes infected cells to innate immune surveillance through altered NK receptor-ligand interactions, while also illustrating the complex balance between activating and inhibitory signalling pathways in EBV immune evasion.

Amarillo et al. explored CD4+ and CD8+ T cell responses in children with primary and persistent EBV infection. Although they demonstrated a higher percentage of activated HLA-DR+ CD8 T cells in children with primary infection, expression of the regulatory molecule, PD-1, was increased in both primary-infected and healthy carriers, in particular on activated HLA-DR+ CD8 T cells, suggesting features of exhaustion. Furthermore, they observed that CD4+ and CD8+ T cells may influence the expression of the latent viral antigens EBNA2 and LMP1 in tonsillar tissue.

To further delineate mechanisms of immune dysregulation during primary Epstein–Barr virus (EBV) infection in children, Mihatsch et al. investigated the role of myeloid-derived suppressor cells (MDSCs) in paediatric patients with infectious mononucleosis (IM). Although the overall frequency of circulating MDSCs did not significantly differ from controls, detailed temporal immunophenotyping revealed dynamic shifts in distinct MDSC-like subsets, including polymorphonuclear-like (PMN-like), monocytic-like (M-like), and early-stage (e-like) MDSCs, across the early and late phases of disease. Importantly, these alterations correlated with both clinical severity and increasing laboratory complexity, including markers of systemic inflammation and immune activation. The findings suggest that specific MDSC subpopulations may contribute to the immunoregulatory environment associated with acute EBV infection and could serve as biomarkers of disease activity and immune dysregulation in EBV-driven disorders.

Two additional reports characterizing the immune response in children and adolescents with EBV-associated diseases were presented. In the first by Ma et al. a 12-year-old child who initially presented with EBV-associated hemophagocytic lymphohistiocytosis (HLH), later progressed to aggressive NK-cell leukemia (ANKL). In this scenario, EBV infection triggered a series of complex T/NK lymphoproliferative disorders that occurred sequentially or simultaneously. In the second report Chen et al. described a case of a previously healthy 19-year-old female who presented with hemophagocytic lymphohistiocytosis (HLH) triggered by EBV infection. They proposed that early recognition of EBV-associated HLH and timely initiation of combined corticosteroid and antiviral therapy might effectively control cytokine storms and improve clinical outcomes in patients.

Xue et al. reported a case of EBV-positive small cell neuroendocrine carcinoma of the nasopharynx with cervical lymph node metastasis, an extremely rare head and neck malignancy.

Bai et al. presented three cases of low-grade malignancy arising from the proliferation of follicular dendritic cells, diagnosed as EBV-positive inflammatory follicular dendritic cell sarcoma (EBV+ IFDCS), a rare condition that primarily arises in the liver and spleen.

Ruiz-Pablos et al. proposed that EBV and other persistent viral infections may contribute to autoimmune inflammation of the pituitary gland in genetically predisposed individuals, leading to secondary hypocortisolism and impaired immune regulation. The authors suggested this failure to control immune hyperactivation may promote chronic inflammation and T-cell exhaustion in myalgic encephalomyelitis/chronic fatigue syndrome (ME/CFS) and related post-viral syndromes, potentially within the spectrum of autoimmune/autoinflammatory syndrome induced by adjuvants (ASIA). They also discussed the potential symptomatic benefits of corticosteroid replacement therapy.

Finally, Mangiaterra et al. presented an extensive review of the mechanisms by which EBV establishes its persistence in the host cell and employs mechanisms of “hit and run”. These mechanisms address the possibility that the virus contributes to the initial steps of cellular transformation and that oncogenic mutations replace the oncogenic function of the virus before it leaves the cell.

Taken together, the contributions to this Research Topic enhance our understanding of the complex interactions between EBV and the immune system, and identify potential therapeutic targets to improve management of EBV-related diseases.

